# NaCl Pretreatment Enhances the Low Temperature Tolerance of Tomato Through Photosynthetic Acclimation

**DOI:** 10.3389/fpls.2022.891697

**Published:** 2022-06-13

**Authors:** Xiaolong Yang, Fengyu Zou, Yumeng Zhang, Jiali Shi, Mingfang Qi, Yufeng Liu, Tianlai Li

**Affiliations:** ^1^Key Laboratory of Protected Horticulture of Ministry of Education, National and Local Joint Engineering Research Center of Northern Horticultural Facilities Design and Application Technology (Liaoning), College of Horticulture, Shenyang Agricultural University, Shenyang, China; ^2^College of Horticulture, South China Agricultural University, Guangzhou, China; ^3^Jiuquan Academy of Agricultural Sciences, Jiuquan, China

**Keywords:** tomato, salt stress, cross-tolerance, cyclic electron transport, non-photochemical quenching, photosynthetic acclimation

## Abstract

Plants often need to withstand multiple types of environmental stresses (e.g., salt and low temperature stress) because of their sessile nature. Although the physiological responses of plants to single stressor have been well-characterized, few studies have evaluated the extent to which pretreatment with non-lethal stressors can maintain the photosynthetic performance of plants in adverse environments (i.e., acclimation-induced cross-tolerance). Here, we studied the effects of sodium chloride (NaCl) pretreatment on the photosynthetic performance of tomato plants exposed to low temperature stress by measuring photosynthetic and chlorophyll fluorescence parameters, stomatal aperture, chloroplast quality, and the expression of stress signaling pathway-related genes. NaCl pretreatment significantly reduced the carbon dioxide assimilation rate, transpiration rate, and stomatal aperture of tomato leaves, but these physiological acclimations could mitigate the adverse effects of subsequent low temperatures compared with non-pretreated tomato plants. The content of photosynthetic pigments decreased and the ultra-microstructure of chloroplasts was damaged under low temperature stress, and the magnitude of these adverse effects was alleviated by NaCl pretreatment. The quantum yield of photosystem I (PSI) and photosystem II (PSII), the quantum yield of regulatory energy dissipation, and non-photochemical energy dissipation owing to donor-side limitation decreased following NaCl treatment; however, the opposite patterns were observed when NaCl-pretreated plants were exposed to low temperature stress. Similar results were obtained for the electron transfer rate of PSI, the electron transfer rate of PSII, and the estimated cyclic electron flow value (CEF). The production of reactive oxygen species induced by low temperature stress was also significantly alleviated by NaCl pretreatment. The expression of ion channel and tubulin-related genes affecting stomatal aperture, chlorophyll synthesis genes, antioxidant enzyme-related genes, and abscisic acid (ABA) and low temperature signaling-related genes was up-regulated in NaCl-pretreated plants under low temperature stress. Our findings indicated that CEF-mediated photoprotection, stomatal movement, the maintenance of chloroplast quality, and ABA and low temperature signaling pathways all play key roles in maintaining the photosynthetic capacity of NaCl-treated tomato plants under low temperature stress.

## Introduction

Because of their sessile nature, plants are often exposed to unfavorable environmental conditions, such as high soil salinity and low temperatures. The abiotic stress has deleterious effects on the photosynthesis efficiency and redistribution the energy from growth to stress resistance, which can drastically decrease crop yield and quality (Zhang et al., 2020). Plants employ a sensitive and complex regulatory system to ensure survival in unpredictable environments (Bailey-Serres et al., 2019; Morales and Kaiser, 2020). Photosynthetic efficiency is maintained in plant leaves through various photoprotective pathways, including the rapid induction and relaxation of non-photochemical quenching (NPQ) and cyclic electron transport around photosystem I (PSI), to prevent oxidative damage to the photosynthetic apparatus caused by reactive oxygen species (ROS) (Pinnola and Bassi, 2018; Park et al., 2019). Soil salinization affects large areas of agricultural land used for crop production worldwide. The main effects of soil salinization on plants include the creation of a hyperosmotic state, which can lead to ion toxicity, and oxidative damage associated with the accumulation of ROS, which can slow growth and result in developmental and metabolic abnormalities (Yang and Guo, 2018; Saddhe et al., 2019). Salt stress induces downstream signaling pathways that trigger a series of cellular responses that mediate the re-establishment of homeostasis and the alleviation of stress-induced damage (Zhao et al., 2020).

Plants often experience multiple abiotic stresses simultaneously or successively; the unique response and specific pathways play a critical role in the acclimation of plants to multifactorial stress combination (Zandalinas et al., 2021). Exposure to a single non-lethal stressor can sometimes confer resistance to various adverse conditions in plants, and this phenomenon is referred to as cross-tolerance (Bowler and Fluhr, 2000). Acclimation to specific stresses in plants is achieved by triggering a regulatory cascade or network that includes the stress stimulus, perception, signal transduction, transcriptional regulation of target genes, and physiological responses (Tombesi et al., 2018). Generally, the action of specific signaling pathways early in the stress response is critically important for the maintenance of cell functions, and common or overlapping signaling pathways and components often act near the end of stress response cascades (Pastori and Foyer, 2002; Locato et al., 2018). The resistance of tomato plants to low temperature and drought stress can be induced by mild low temperature, paraquat, and drought pretreatment, and this cross-tolerance mechanism involves the activation of ROS-mediated signal transduction pathways (Zhou et al., 2014). Pretreatment of soil with salt has been shown to result in higher leaf mass per area, total chlorophyll (Chl) and carotenoid (Car) content, and photosynthetic activity in tomato plants fumigated with sulfur (Jiang et al., 2017). In addition, drought pretreatment can induce resistance to heat in tall fescue and Arabidopsis, and heat shock and NaCl treatment can induce resistance to UV-B radiation in barley (Çakirlar et al., 2008; Zhang et al., 2019).

A particularly effective strategy for improving crop yields under abiotic stress is to enhance the photosynthetic capacity of crops (Gururani et al., 2015). Extensive studies have characterized the effects of single stressors on photosynthesis using plant genetic engineering techniques and photosynthetic fluorescence analysis (Guidi et al., 2019). Soil salinity pretreatment can alleviate the damage to photosynthetic capacity induced by drought treatment in tomato plants; however, the cross-tolerance mechanism mediating the photosynthetic capacity response remains unclear (Yang et al., 2020). Salt stress is a very common abiotic stress in vegetable production, especially the accumulation of salt in the soil surface due to the frequent irrigation. In addition, plants are still hard to avoid the adverse effects of low temperature even growth in energy-saving solar greenhouse in northern China. Salt stress and low temperature are considered to be the major factors limiting vegetable production to a certain extent, there is thus a need to determine how exposure to soil salinity affects the tradeoff between photoprotection, photochemistry, and chloroplast quality and confers tolerance to low temperature stress. The aim of this study was to explore the photosynthetic performance of tomato plants pretreated with sodium chloride (NaCl) under low temperature stress. Generally, the results of this study provide new insights that enhance our understanding of acclimation-induced cross-tolerance and have implications for environmental management during vegetable production.

## Materials and Methods

### Plant Materials and Treatments

Experiments were conducted in the solar climate chamber at Shenyang Agricultural University from May to October 2019. The tomato (*Solanum lycopersicum* L.) variety “Liao Yuan Duo Li” was used in experiments, and seeds were germinated in seedling trays and transferred to plastic pots at the two-leaf stage. The growth temperature was controlled at approximately 25/15°C (day/night, 12 h/12 h), the humidity was ~50% during the day and 80% at night, and the light intensity was approximately 800 μmol·photons·m^−2^·s^−1^ natural solar radiation at noon. Before the experiment, ~50–100 mL of water was applied to each seedling per day to ensure consistent growth. Plants were exposed to the NaCl pretreatment and the low temperature stress treatment when they had reached the five-leaf stage. For the NaCl pretreatment, 100 mL of water and 100 mL of 100 mM NaCl solution were applied every morning for 5 days. The plants were then divided into the normal temperature group (CK, NaCl) and low temperature group (CK+LT, NaCl+LT). Plants in the normal temperature group were exposed to 25/15°C (day/night) for 5 days, and plants in the low temperature group were exposed to 15/6°C for 5 days. Measurements were taken on the first (T1) and fifth (T5) day after NaCl pretreatment and the fifth day (T10) after low temperature treatment.

### Measurement of Leaf Photosynthesis Gas Exchange

A synchronous measurement system with a GFS-3000 photosynthesizer and Dual-PAM-100 fluorescence analyzer (Heinz Walz, Effeltrich, Germany) was used to analyze the photosynthetic gas exchange and Chl fluorescence of plant leaves *in vivo* using standard measurement procedures and settings but with various modifications (Zhang et al., 2014; Lu et al., 2017; Yang et al., 2018, 2020). All measurements were made using the fourth functional leaf from the top of each plant; the area of the measuring head was 1.3 cm^2^. During measurements, the air inlet of the photosynthetic apparatus was connected to a 10-L air buffer bottle so that the ambient atmospheric carbon dioxide (CO_2_) concentration could be taken as a reference; the indoor temperature was approximately 25°C, and the light intensity was 1,100 μmol·photos·m^−2^·s^−1^. When leaf photosynthesis gas exchange reached a stable state after full photoadaptation, the net photosynthetic rate (Pn), intercellular CO_2_ concentration (Ci), stomatal conductance (GH_2_O), transpiration rate (E), water use efficiency (WUE), stomatal limit value (*L*s), and other parameters were measured.

### Measurement of Pigment Content and Observations of Stomatal Aperture

The content of photosynthetic pigments in tomato leaves was determined by the ethanol and acetone extraction method. Specifically, 0.2 g of fresh leaf samples were placed into a 20-mL test tube; 10 mL of a 1:1 mixture of 95% ethanol and 80% acetone was then added, and the mixture was left to stand in a dark environment for 24 h. The optical density (OD) was measured using a UV 1200 ultraviolet spectrophotometer (Shimadzu, Kyoto, Japan), and calculated by the following equations: the content of Chlorophyll a (mg·g^−1^) = (12.72 OD663–2.59 OD645) V/ 1,000 W; the content of Chlorophyll b (mg·g^−1^) = (22.88 OD645–4.67 OD663) V/ 1,000 W; the content of Carotenoid (mg·g^−1^) = (1,000 OD470–3.27 Chl a – 104 Chl b) V/ (229 × 1,000 W). Where V is the total volume of ethanol and acetone extract (mL), and W is the fresh weight (g) of the sample (Fan et al., 2013; Yang et al., 2018). The lower epidermis of tomato was removed with tweezers and placed on a microscope slide, and appropriate distilled water was placed on the slide to ensure that samples were completely immersed in fluid. Each treatment was repeated six times. Observations and photography of the stomata aperture were carried out using a fluorescence inverted microscope (Axio Observer A1, Zeiss, Germany); 10 photographs of each leaf were taken at random times for measurements of stomatal parameters.

### Chloroplast Ultramicrostructure Observations

Veinless strips (1 × 2 mm) from tomato leaves were fixed with 2.5% glutaraldehyde and 1% acetic acid and dehydrated with ethanol; they were then embedded with epoxy resin, sliced, and stained (Hao et al., 2016). An ultra-thin slicing machine (Leica EM UC7, Germany) was used to make ultra-thin slices. The ultramicrostructure of the chloroplast was observed and photographed using transmission electron microscope (Hitachi HT-7700, Japan) under 1,500 ×, 6,000 ×, and 20,000 × magnification. Ten photos were taken of each sample for measurements of chloroplast ultramicrostructure parameters.

### Measurement of the OJIP Induction Curve and the P700 Redox Status

A saturation pulse (300 ms, 10,000 μmol·photons·m^−2^·s^−1^) was used to determine the OJIP induction curve of the Chl a fluorescence per the automated routines provided by Dual-PAM software following dark adaptation for at least 30 min (Zhang et al., 2014; Lu et al., 2017; Yang et al., 2018, 2020). The redox state of P700 was determined *in vivo* using the dual-beam 870–830 nm signal difference provided by the Dual-PAM-100 system. Single-turnover flash (ST, 50 ms) induction of the oxidation of PQ pools and multiple-turnover flash (MT, 50 ms) induction of the full reduction of PQ pools in the presence of far-red light were used to measure the redox kinetics of P700. The complementary areas of ST and MT excitation signal change were used to calculate the functional pool sizes of intersystem electrons on a P700 reaction center as follows: PQ size = MT-areas/ST-areas (Zhang et al., 2014; Lu et al., 2017; Yang et al., 2018, 2020).

### Measurement of Light Energy Conversion and the Electron Transfer Rate

All measurements were conducted on plants following dark adaptation for more than 30 min. The slow Chl fluorescence induction curve was then recorded for 520 s. A low intensity measuring light was used to detect the minimum fluorescence, F0; a saturating pulse (10,000 μmol·photons·m^−2^·s^−1^) was then applied to detect the maximum fluorescence, Fm. A saturation pulse after illumination with far-red light was used to measure the maximum change in the P700 signal, Pm. A saturating pulse (300 ms, 10,000 μmol·photons·m^−2^·s^−1^) was applied every 20 s after the actinic light (191 μmol·photons·m^−2^·s^−1^, 635 nm) was turned on to determine the maximum fluorescence signal (Fm′) and maximum P700^+^ signal (Pm′) under light adaptation for 8 min. The rapid light response curves (RLCs) were determined using the standard measurement program immediately after slow induction curve measurements. The light intensity of the RLC changed every 30 s in the sequence 29, 37, 55, 113, 191, 213, 349, 520, 778, 1,197, and 1,474 μmol·photons·m^−2^·s^−1^, and a saturating pulse was used to measure Fm′ and Pm′ after each period of actinic light. The parameters measured in this study were as follows: maximum photochemical quantum yield of PSII, Fv/Fm; effective quantum yield of PSII, Y(II); quantum yield of non-regulatory energy dissipation, Y(NO); quantum yield of regulatory energy dissipation, Y(NPQ); non-photochemical quenching in PSII, NPQ; quantum yield of PSI, Y(I); quantum yield of non-photochemical energy dissipation owing to acceptor-side limitation, Y(NA); quantum yield of PSI non-photochemical energy dissipation owing to donor-side limitation, Y(ND); electron transfer rate of PSI, ETR(I); electron transfer rate of PSII, ETR(II); and estimated cyclic electron flow value (CEF), which was determined by ETR(I)–ETR(II) (Zhang et al., 2014; Lu et al., 2017; Yang et al., 2018, 2020; Sun et al., 2022).

### Analysis of ROS Production and Antioxidant Enzyme Activity

The O${}_{2}^{-}$ production rate and H_2_O_2_ content were determined by the hydroxylamine oxidation method (Ibrahim and Jaafar, 2012; Lu et al., 2020b). Briefly, 0.2 g of tomato leaves were placed in a mortar, and 3 mL of 50 mM PBS buffer (pH 7.8) was added three times; the mixture was then fully ground and centrifuged at 10,000 g and 4°C for 20 min, and the supernatant, which comprised the enzyme extract, was collected. The methods of Lu (2020) were used to determine the superoxide dismutase (SOD) and peroxidase (POD) activities.

### Quantitative Real-Time Polymerase Chain Reaction

Fresh leaf samples (0.2 g) were taken, quick-frozen with liquid nitrogen, and then stored at −80°C. Total RNA was extracted per the instructions of the RNA extraction kit (Kangwei, Biotech, Beijing, China). The quality of RNA was evaluated using 1% agarose gel electrophoresis, and the concentration and purity (28SrRNA/18SrRNA) were measured using a NanoDrop spectrophotometer ND-1000 (NanoDrop, USA). 1 μg of RNA was reverse-transcribed into cDNA using Prime ScriptTM RT Master Mix (Perfect Real Time, Takara) and stored at −20°C. Real-time quantitative fluorescence polymerase chain reaction (PCR) was conducted following the instructions in the Super Real PreMix Plus (SYBR Green) (TaKaRa, Dalian, China) kit, and the amplification procedure was conducted using a real-time quantitative fluorescence PCR instrument; the primers used are shown in Supplementary Table 1.

### Statistical Analysis

Student's *t*-tests were conducted in SPSS version 22 (SPSS, Armonk, NY, USA) to evaluate the significance of differences between treatments. The mean values of three to six independent biological replicates were calculated and presented as mean ± standard deviation (SD), and the threshold for significance was *P* ≤ 0.05. All graphs were made using Origin Version 12.0 (Systat, San Jose, CA, USA).

## Results

### Stomatal Aperture and Photosynthetic Gas Exchange in Tomato Leaves

**Figure 1:**
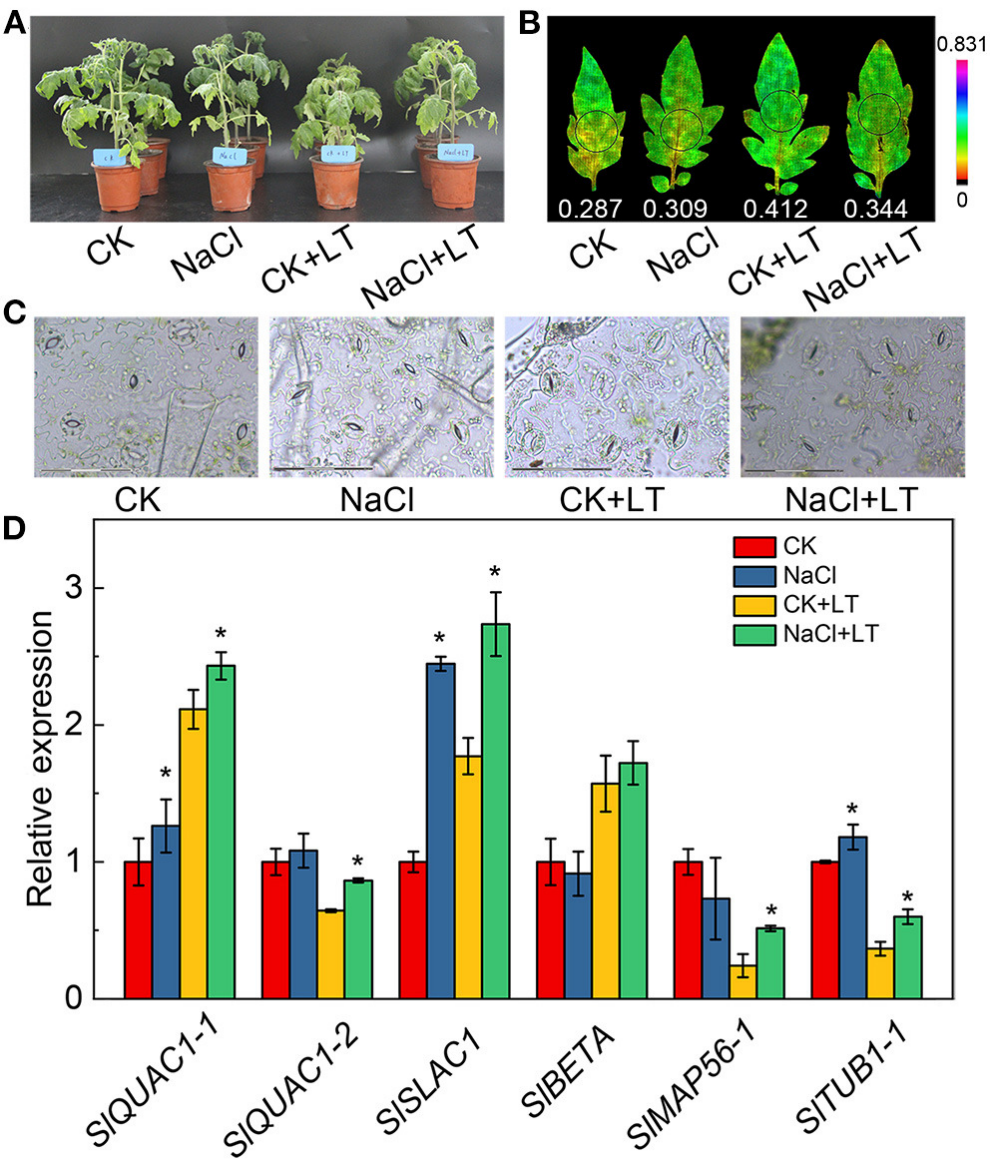
Effects of NaCl pretreatment on the phenotype, stomatal movements, and expression of ion transport-related genes of tomato leaves under low temperature treatment. The growth state of tomato plants under different treatments at different stages **(A)**; Chl fluorescence imaging of Y(NPQ) **(B)**; images of the stomata on tomato leaves under different treatments **(C)**; and relative expression of ion channel and tubulin-related genes **(D)**. The results are shown as mean values of three to five independent biological replicates ± SD, * indicate significant differences between CK and NaCl and between CK+LT and NaCl+LT (*P* < 0.05, Student's *t*-test).

**Figure 2:**
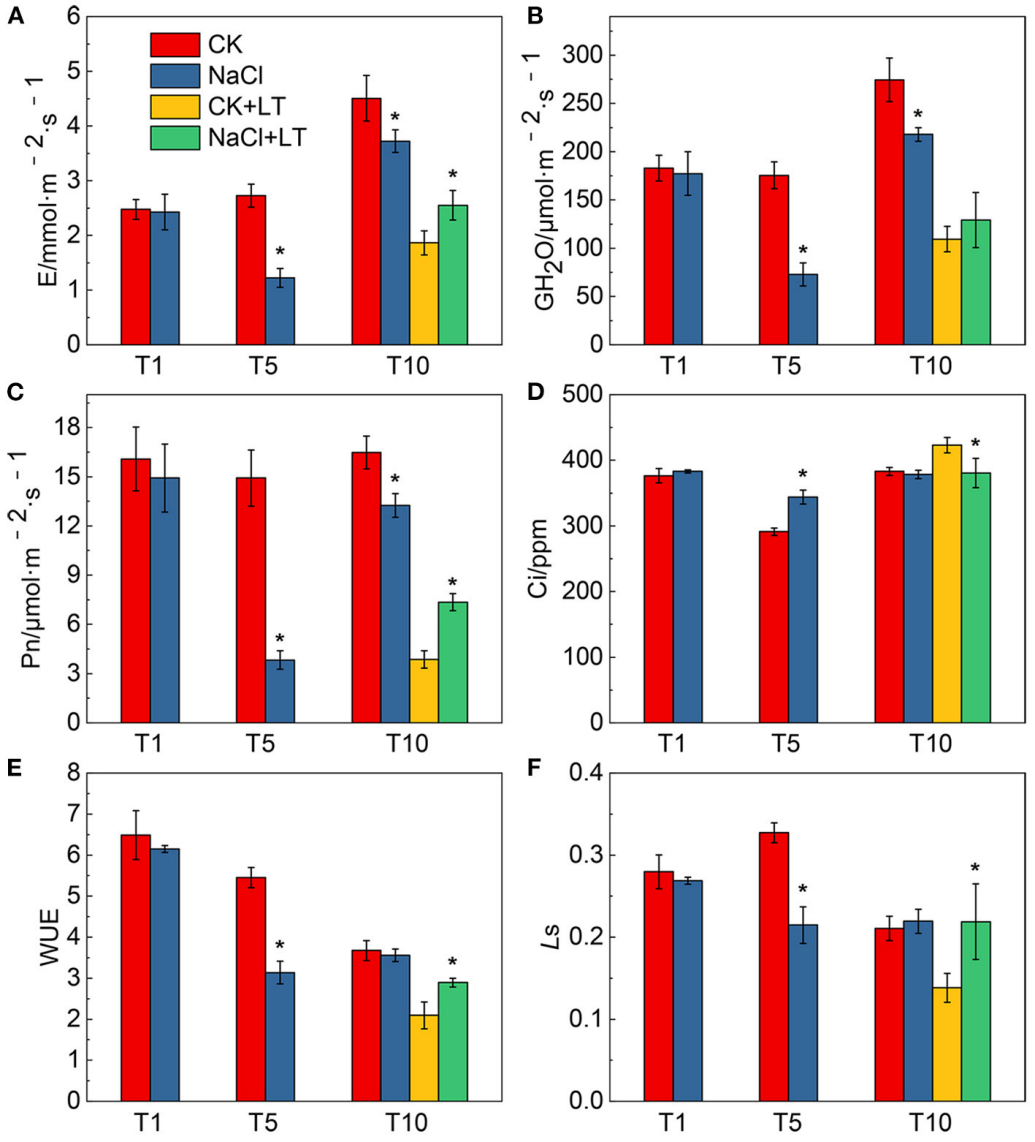
Effects of NaCl pretreatment on the photosynthetic gas exchange parameters of tomato leaves under low temperature stress. The effect of NaCl pretreatment on the transpiration rate **(A,E)**, stomatal conductance (GH_2_O) **(B)**, net photosynthetic rate (Pn) **(C)**, intercellular CO_2_ concentration (Ci) **(D)**, water use efficiency (WUE) **(E)**, and stomatal limitation value (*L*s) **(F)** in tomato leaves under low temperature stress. The results are shown as the mean values of six independent biological replicates ± SD, * indicate significant differences between CK and NaCl and between CK+LT and NaCl+LT (*P* < 0.05, Student's *t*-test).

The growth potential of NaCl-pretreated plants was greater than that of plants in the CK+LT treatment under low temperature stress (Figure 1A). The stomatal width of tomato leaves was significantly reduced by NaCl treatment at room temperature and under low temperature treatment; however, NaCl+LT treatment significantly increased the stomatal width and reduced the stomatal length and the ratio of Length/Width compared with CK+LT treatment (Figure 1C, Table 1). The relative expression levels of the ion channel-related genes *SlQuAC1-1, SlQuAC1-2* and *SlSLAC1* and tubulin-related genes *SlMAP56-1* and *SlTUB1-1* were significantly up-regulated at low temperatures in NaCl-pretreated plants compared with plants in the CK+LT treatment (Figure 1D). These findings indicate that NaCl pretreatment affects the expression of ion channel and tubulin-related genes in leaves at low temperature, which might affect the concentrations of ions inside and outside the guard cells and thus stomatal opening. The photosynthesis gas exchange parameters have no significantly differences between treatments at T1; NaCl-pretreated significantly reduced the parameters E, Pn, *g*H_2_O, Ls, and WUE of plants than CK at T5. After 5 days of low temperature treatment, E, Pn, *g*H_2_O, and WUE were significantly higher in NaCl-treated plants (NaCl+LT) than in plants in the CK+LT treatment, indicating that NaCl pretreatment enhances the CO_2_ assimilation efficiency under low temperature stress (Figure 2). This result might be related to the effect of NaCl pretreatment on the regulation of stomatal opening and the water use of plants.

**Table 1:** Effects of NaCl pretreatment on the stomatal aperture of tomato leaves under low temperature stress.

**Treatments**	**Length (μm)**	**Width (μm)**	**Length/width**
CK	11.27 ± 0.43a	5.61 ± 0.81a	2.03 ± 0.23d
NaCl	11.72 ± 0.27a	3.46 ± 0.18b	3.39 ± 0.18c
CK+LT	11.30 ± 0.20a	2.49 ± 0.18d	4.55 ± 0.26a
NaCl+LT	10.59 ± 0.47b	2.97 ± 0.63c	3.72 ± 1.04b

*The results are shown as mean values of six independent biological replicates ± SD, different letters in the same column indicate significant differences according to Student's t-test (P < 0.05)*.

### Chl Metabolism and Chloroplast Ultramicrostructure

**Figure 3:**
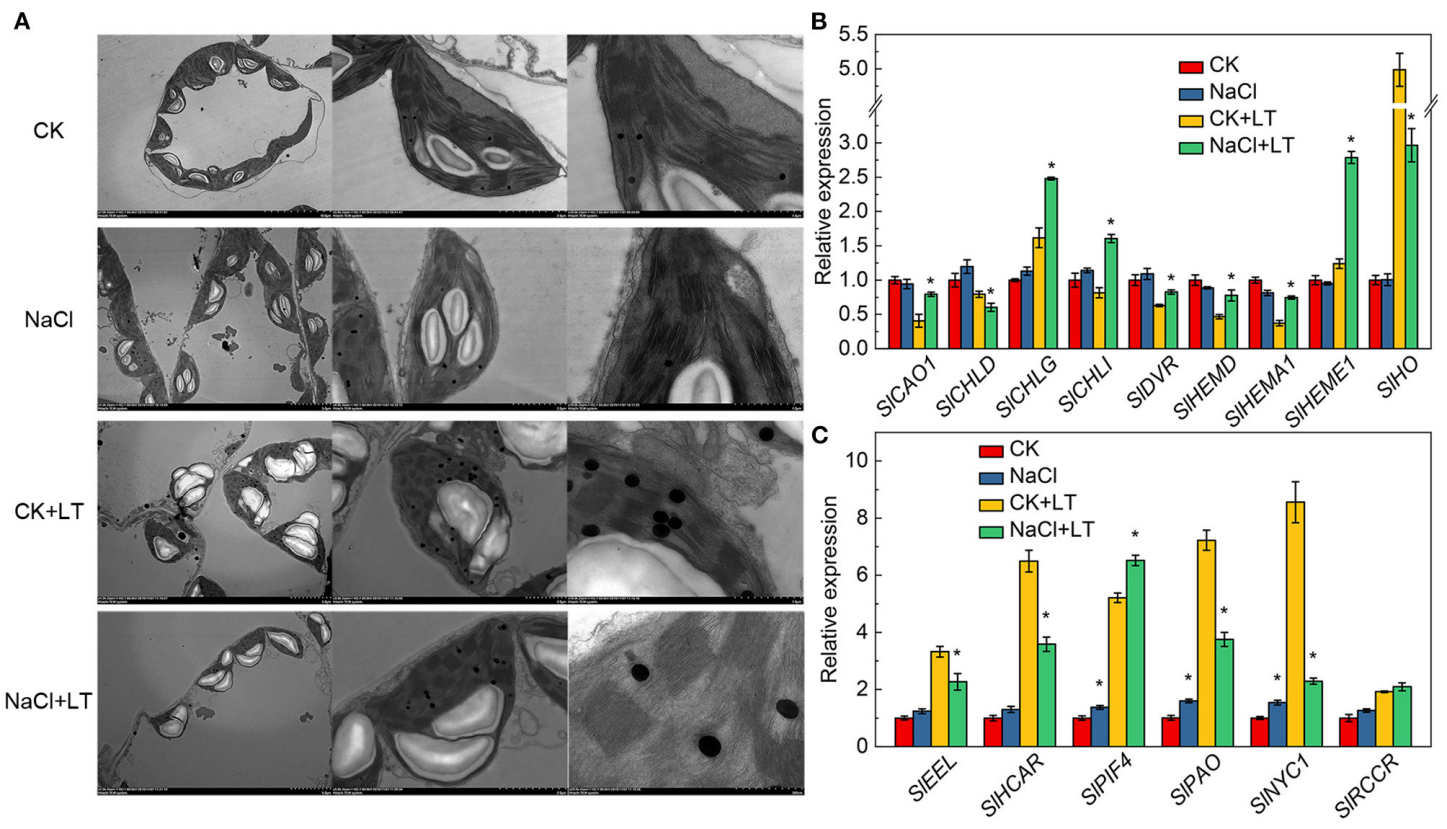
Effects of NaCl pretreatment on the ultramicrostructure of chloroplasts and expression of genes associated with chlorophyll metabolism of tomato leaves under low temperature stress. The ultramicrostructure of chloroplasts **(A)**, the expression of Chl synthesis **(B)** and decomposition pathway-related genes **(C)** in tomato leaves. The results are shown as the mean values of three independent biological replicates ± SD, * indicate significant differences between CK and NaCl and between CK+LT and NaCl+LT (*P* < 0.05, Student's *t*-test).

The ultramicrostructure of the chloroplasts was not significantly affected by the NaCl treatment. However, low temperature stress resulted in the destruction of the chloroplast membrane, significantly reduced the chloroplast length-to-width ratio, and significantly increased the number of starch grains per chloroplast. In the NaCl+LT treatment, the ultramicrostructure of the chloroplasts was more complete, the chloroplast membrane was intact, the crenellate structure of the thylakoid was clear, the chloroplast length-to-width ratio was significantly increased, and the number of starch grains and glutathione grains in chloroplasts was significantly reduced compared with the CK+LT treatment (Figure 3A, Table 2). These results suggested that NaCl pretreatment might enhance the photosynthetic capacity of tomato by alleviating the damage of low temperature on chloroplast structure. Low temperature treatment significantly reduced the content of photosynthetic pigments, and the content of Chl *a*, Chl *b*, Car, and total Chl was significantly higher in the NaCl+LT treatment than in the CK+LT treatment (Table 3). The relative expression levels of the Chl synthesis genes *SlCAO1, SlCHLG, SlCHLI, SlDVR, SlHEMD, SlHEMA1*, and *SlHEME1* were significantly up-regulated by NaCl pretreatment; in addition, the relative expression levels of the Chl decomposition-related genes *SlEEL, SlHCAR, SlPAO*, and *SlNYC1* were significantly down-regulated under low temperatures stress compared with the CK+LT treatment (Figures 3B,C). These findings indicate that NaCl pretreatment can maintain the content of photosynthetic pigments by enhancing the photosynthetic capacity of tomato under low temperature stress.

**Table 2:** Effect of NaCl pretreatment on the chloroplast ultramicrostructure in tomato leaves under low temperature treatment.

**Types**	**Chloroplast length/μm**	**Chloroplast width/μm**	**Chloroplast length/width**
CK	5.47 ± 0.21a	2.86 ± 0.07c	1.94 ± 0.05a
NaCl	5.37 ± 0.22a	2.92 ± 0.06c	1.84 ± 0.06a
LT	4.28 ± 0.06c	3.43 ± 0.08a	1.25 ± 0.04c
LT+NaCl	4.81 ± 0.05b	3.18 ± 0.05b	1.51 ± 0.04b

*The results are shown as mean values of three independent biological replicates ± SD, different letters in the same column indicate significant differences according to Student's t-test (P < 0.05)*.

**Table 3:** Effect of NaCl pretreatment on the Chl content in tomato leaves under low temperature treatment.

**Types**	**Chlorophyll a /mg·g^**−1**^FW**	**Chlorophyll b** **/mg·g^**−1**^FW**	**Carotenoid /mg·g^**−1**^FW**
CK	8.08 ± 0.10a	1.89 ± 0.03a	2.13 ± 0.07a
NaCl	7.80 ± 0.04b	1.80 ± 0.01ab	2.06 ± 0.01a
LT	6.37 ± 0.05d	1.41 ± 0.02c	1.78 ± 0.01b
LT+NaCl	7.30 ± 0.03c	1.64 ± 0.01b	1.94 ± 0.01ab

*The results are shown as mean values of six independent biological replicates ± SD, different letters in the same column indicate significant differences according to Student's t-test (P < 0.05)*.

### Photochemical Efficiency of PSI and PSII

**Figure 4:**
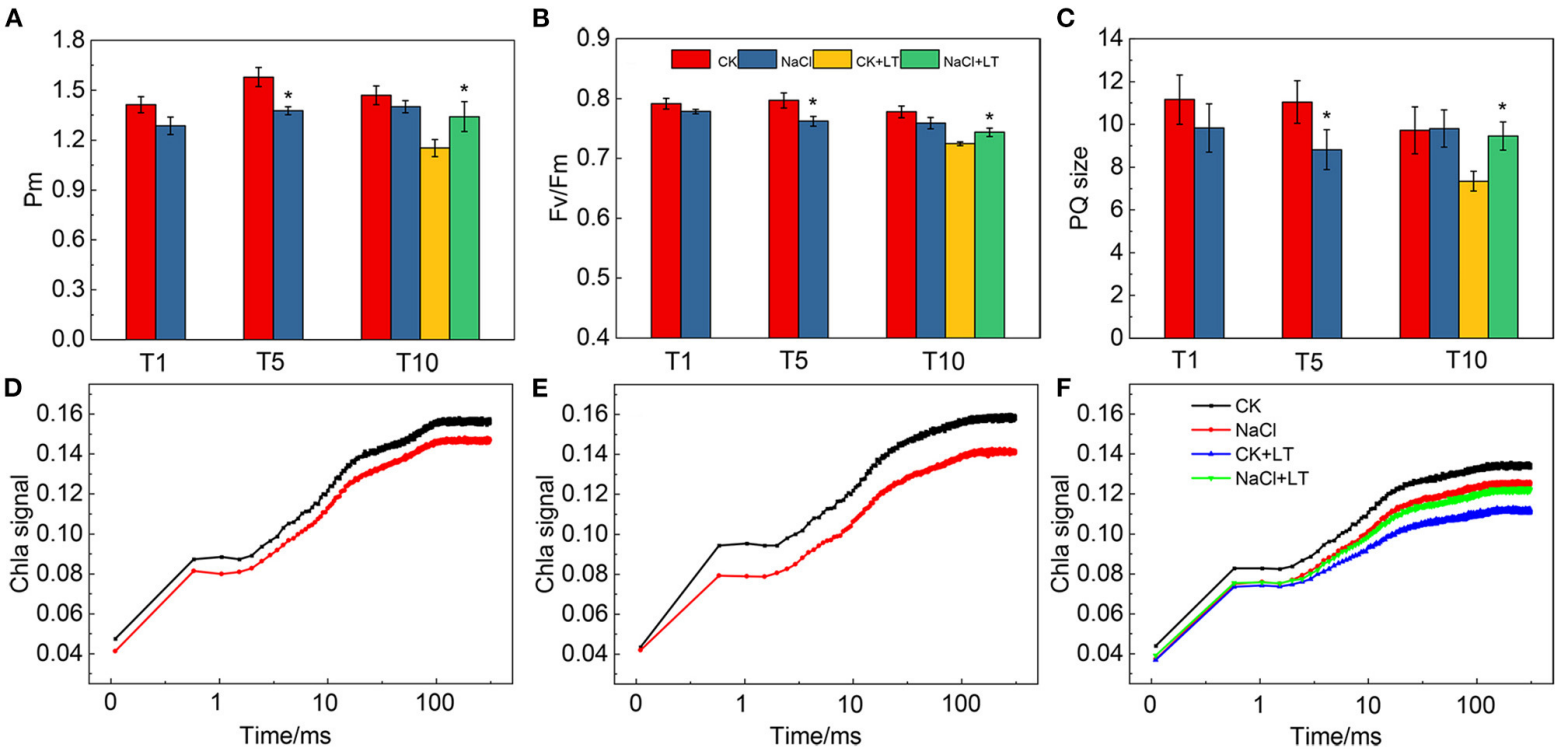
The effect of soil NaCl pretreatment on the photosynthetic activity of PSI and PSII of tomato leaves under low temperature treatment. The maximal redox state of PSI (Pm) **(A)**, the maximum quantum efficiency of PSII (Fv/Fm) **(B)**, the PQ size **(C)**, and the fast induction curve of Chl a fluorescence at T1 **(D)**, T5 **(E)** and T10 **(F)** of tomato leaves. The results are presented as the mean values of five independent biological replicates ± SD, * indicate significant differences between CK and NaCl and between CK+LT and NaCl+LT (*P* < 0.05, Student's *t*-test).

Pm reflects the maximum oxidation state that P700 can reach in the PSI reaction center of leaves; it thus reflects the activity of PSI to a certain extent. Pm was sensitive to NaCl treatment, the results shown 5 days NaCl treatment significantly reduced Pm of tomato leaves compared with CK, while it was significantly lower in CK+LT than in NaCl+LT at T10 (Figure 4A). The results of Fv/Fm were similar to Pm, indicating that NaCl pre-treatment can alleviate the damage to PSI and PSII induced by subsequent low temperature stress (Figure 4B). The ratio between the area of the MT flash/ST flash was used for determination of the relative functional pool size of the intersystem electrons able to reduce PSI reaction center (P700^+^), the results shown PQ size was significantly lower in NaCl-pretreated plants than in CK plants at T5; at T10, the PQ size was significantly lower in plants in the CK+LT treatment than in plants in the NaCl+LT treatment, indicating that NaCl pretreatment can alleviate the deleterious effects of low temperature stress on PQ electron carriers and maintain high electron transport capacity (Figure 4C). The OJIP kinetics curve of leaves under NaCl treatment decreased slightly at T1 and significantly at T5, indicating that NaCl treatment had an effect on the electron transfer of the donor and acceptor sides of PSII. Following low temperature stress, the signal intensity significantly decreased in the CK+LT treatment; the OJIP signal remained strong in the NaCl+LT treatment relative to the CK+LT treatment, suggesting that NaCl pretreatment can alleviate the damage to the PSII reaction center induced by low temperature stress (Figures 4D–F).

### Light Energy Distribution in PSI and PSII

**Figure 5:**
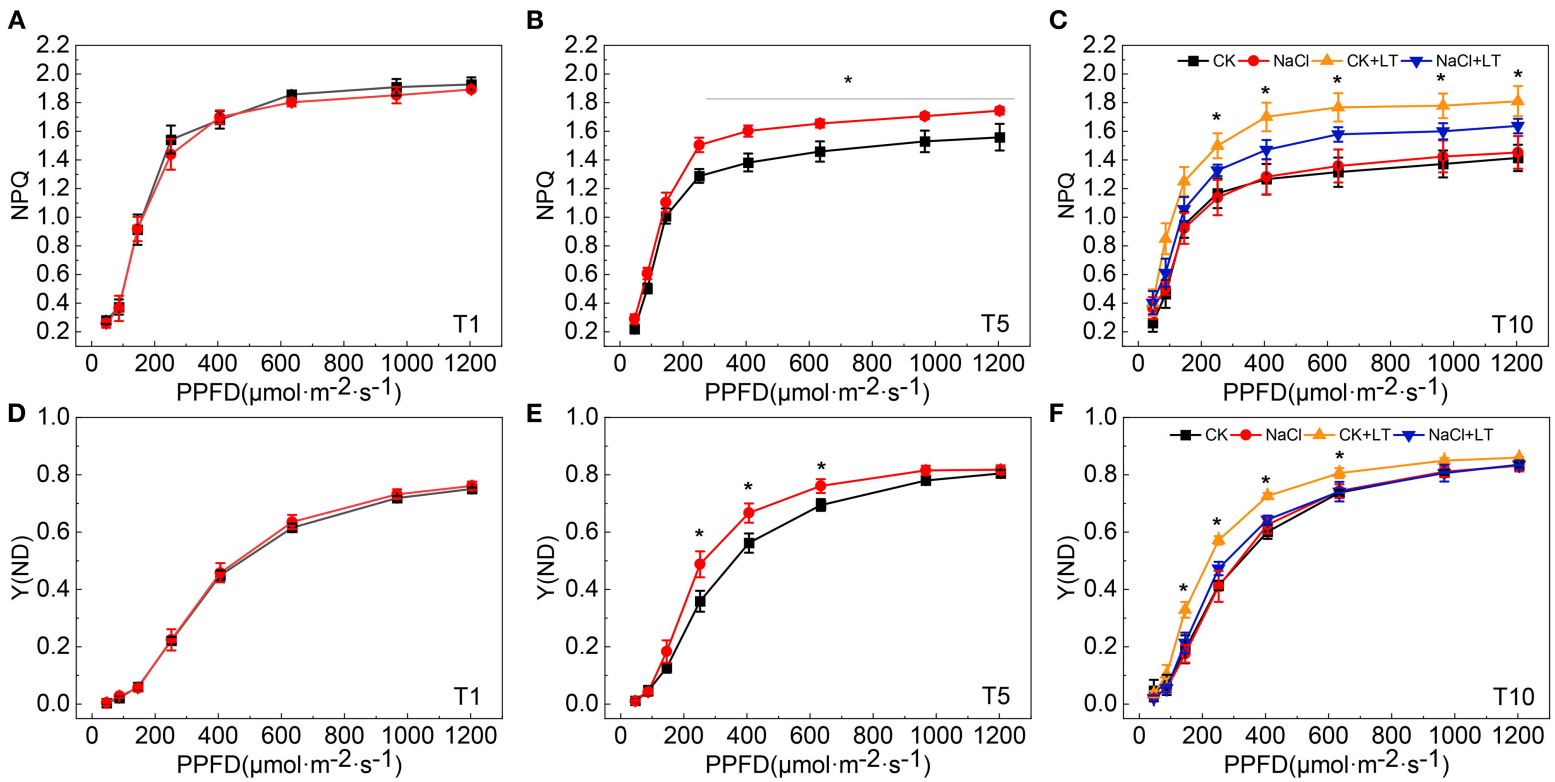
Effect of soil NaCl pretreatment on the rapid light response curve of the NPQ and Y(ND) of tomato leaves under low temperature stress. NPQ at T1 **(A)**; NPQ at T5 **(B)**; NPQ at T10 **(C)**; Y(ND) at T1 **(D)**; Y(ND) at T5 **(E)**; and Y(ND) at T10 **(F)**. The results are presented as the mean values of five independent biological replicates ± SD, * indicate significant differences between CK and NaCl and between CK+LT and NaCl+LT (*P* < 0.05, Student's *t*-test).

**Figure 6:**
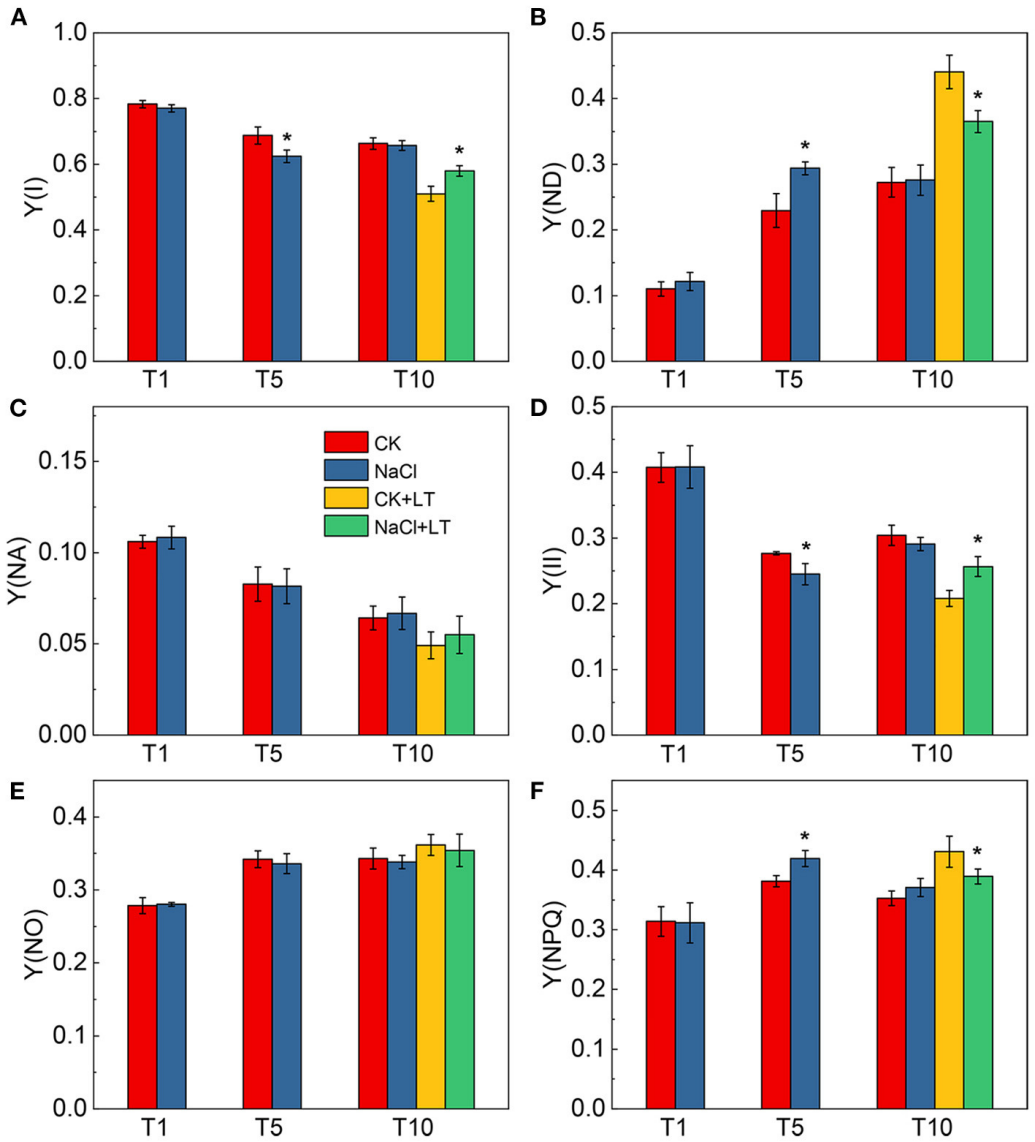
Effect of soil NaCl pretreatment on the energy conversion in PSI and PSII of tomato leaves under drought stress. Y(I) **(A)**; Y(ND) **(B)**; Y(NA) **(C)**; Y(II) **(D)**; Y(NO) **(E)**; and Y(NPQ) **(F)**. The results are presented as the mean values of five independent biological replicates ± SD, * indicate significant differences between CK and NaCl and between CK+LT and NaCl+LT (*P* < 0.05, Student's *t*-test).

When plants are subjected to stress, the excessive light energy absorbed can be dissipated in the form of heat to protect the photosystems from damage. The NPQ of the antenna pigments in PSII and the energy dissipation on the PSI donor side are important regulatory strategies. Both NPQ and Y(ND) increased as the light intensity increase, at T1, NPQ and Y(ND) did not significantly differ between NaCl-pretreated leaves and control leaves; at T5, the NPQ and Y(ND) were significantly higher in NaCl-pretreated leaves than in CK leaves under high light intensity. The NPQ and Y(ND) were significantly higher in the CK+LT treatment than in the NaCl+LT treatment following low temperature stress (Figure 5). The distribution of captured light energy between photosystems plays an important role in regulating the photochemical reactions of photosynthesis. At T1, the fluorescence parameters did not differ between NaCl-pretreated leaves and CK leaves. At T5, Y(I) and Y(II) were significantly reduced and Y(ND) and Y(NPQ) were significantly increased under NaCl pretreatment, and no differences were observed in Y(NA) and Y(NO) among treatments. After exposure to low temperature stress, Y(I) and Y(II) decreased rapidly and were significantly lower in the CK+LT treatment than in the NaCl+LT treatment; Y(NPQ) and Y(ND) increased rapidly and were significantly higher in the CK+LT treatment than in the NaCl+LT treatment following low temperature exposure, indicating that NaCl pretreatment increased the photochemical reaction efficiency of PSI and PSII (Figures 1B, 6).

### Photosynthetic Linear and Cyclic Electron Transport in Tomato Leaves

**Figure 7:**
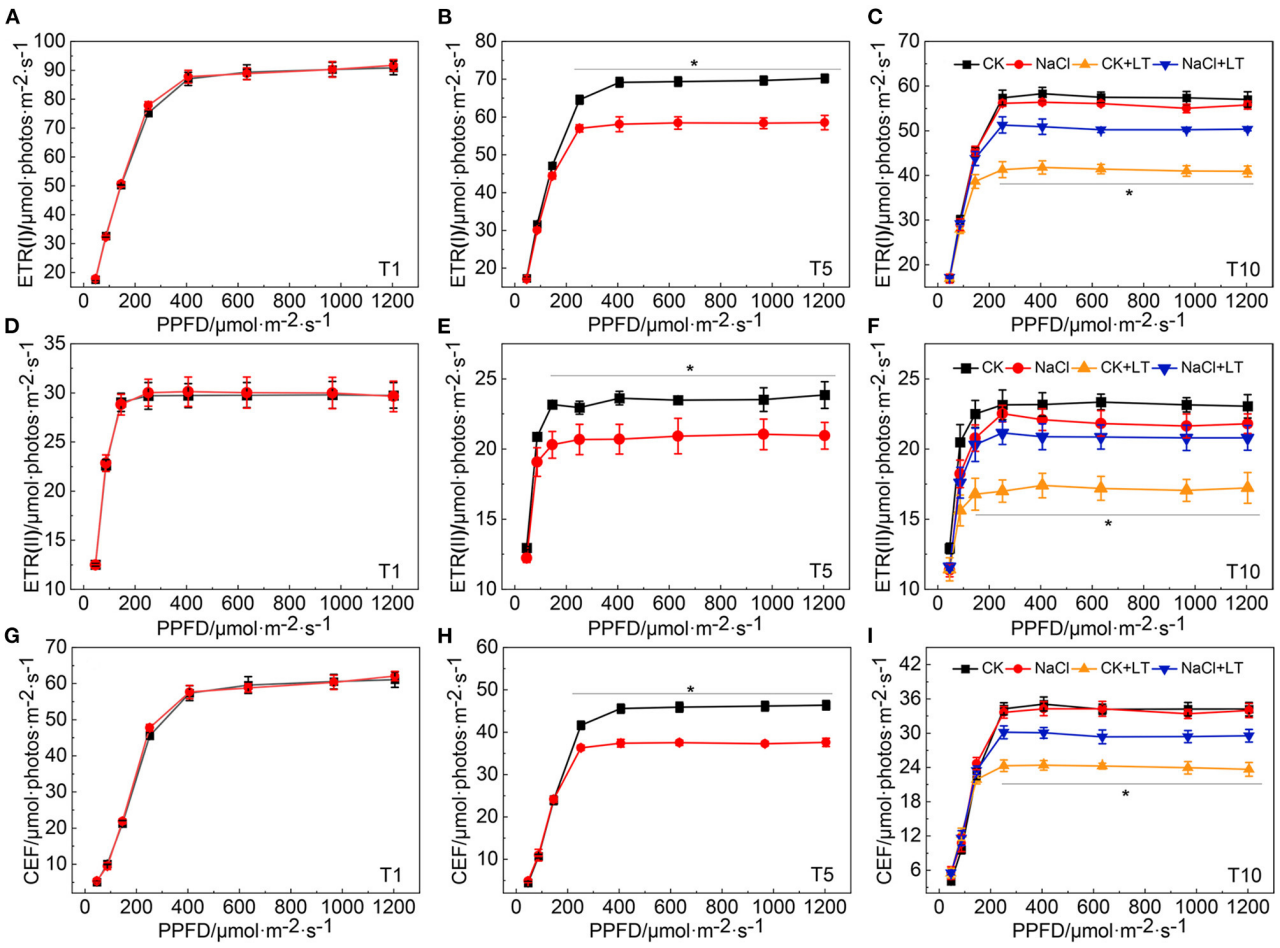
Response of photosynthetic linear and cyclic electron transfer in NaCl-pretreated tomato leaves under low temperature stress. ETR(I) at T1 **(A)**; ETR(I) at T5 **(B)**; and ETR(I) at T10 **(C)**; ETR (II) at T1 **(D)**; ETR (II) at T5 **(E)**; ETR (II) at T10 **(F)**; CEF at T1 **(G)**; CEF at T5 **(H)**; CEF at T10 **(I)**. The results are presented as the mean values of five independent biological replicates ± SD, * indicate significant differences between CK and NaCl and between CK+LT and NaCl+LT (*P* < 0.05, Student's *t*-test).

Analysis of the light intensity-dependent linear and cyclic electron transport rate revealed that the ETR(I), ETR(II), and CEF increased rapidly as the light intensity increased. At T1, there was no difference between treatments. At T5, when the electron transfer rate reached a steady-state, ETR(I), ETR(II), and CEF were significantly lower in NaCl-pretreated leaves than in control leaves, indicating that NaCl treatment reduced both the photosynthetic linear and cyclic electron transfer rate of tomato leaves (Figure 7). The linear and cyclic electron transport rate of plants subjected to low temperature stress were lower than those not subjected to low temperature stress, and the electron transfer rate was significantly higher in NaCl-pretreated leaves than in leaves subjected to low temperature stress that had not been pretreated with NaCl. These findings indicate that despite the reduction in the electron transfer rate caused by NaCl pretreatment, the linear and cyclic electron transport rate remained high under low temperature stress, and these patterns were consistent with the phenotypes observed in each treatment.

### ROS Metabolism and Antioxidant Enzyme Activity

**Figure 8:**
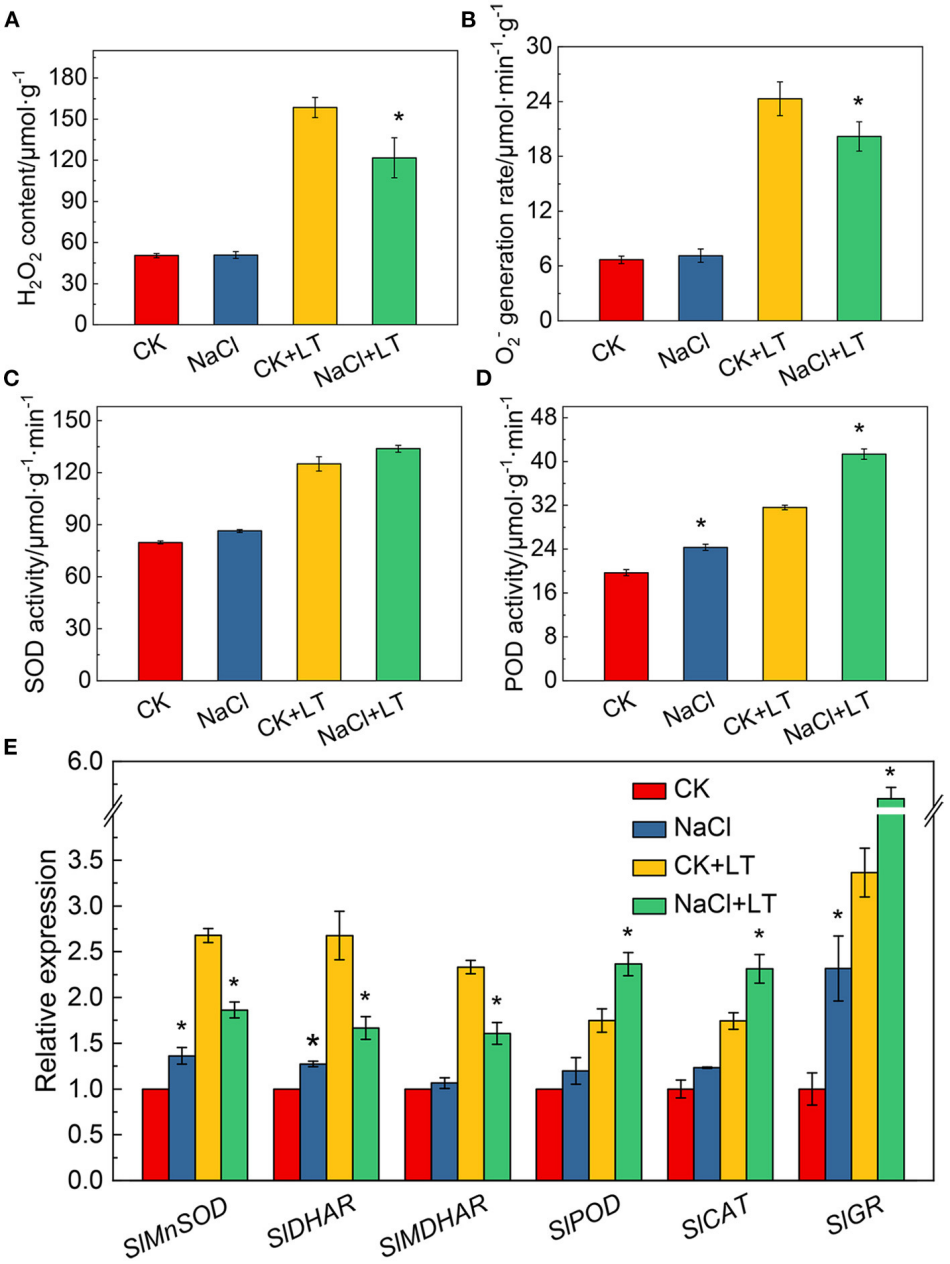
Response of reactive oxygen species production, antioxidant enzyme activity, and the expression of related genes in tomato leaves to salt pretreatment and low temperature stress. H_2_O_2_ content **(A)**; O^2^– generation rate **(B)**; superoxide dismutase (SOD) activity **(C)**; peroxidase (POD) activity **(D)**; and antioxidase-related gene expression **(E)**. The results are presented as the mean values of three independent biological replicates ± SD, * indicate significant differences between CK and NaCl and between CK+LT and NaCl+LT (*P* < 0.05, Student's *t*-test).

The content of H_2_O_2_ and O${}_{2}^{-}$ production rate was greatly increased after low temperature treatment, while it was significantly alleviated in the NaCl+LT treatment compared with the CK+LT treatment, this indicates that NaCl pretreatment could significantly reduce the accumulation of ROS in tomato plants under low temperature stress (Figures 8A,B). POD activity was significantly increased in tomato leaves in the NaCl+LT treatment compared with the CK+LT treatment, but no difference was observed in SOD activity (Figures 8C,D). NaCl pretreatment significantly increased the relative expression levels of *SlPOD, SlCAT*, and *SlGR* in tomato leaves under low temperature stress, indicating that NaCl pretreatment could increase the expression of antioxidase-related genes under low temperature stress. By contrast, the relative expression levels of *SlMnSOD, SlDHAR*, and *SlMDHAR* were significantly down-regulated by NaCl pretreatment under low temperature stress (Figure 8E).

### Expression of ABA Signaling Pathway and Cold Stress-Related Genes

**Figure 9:**
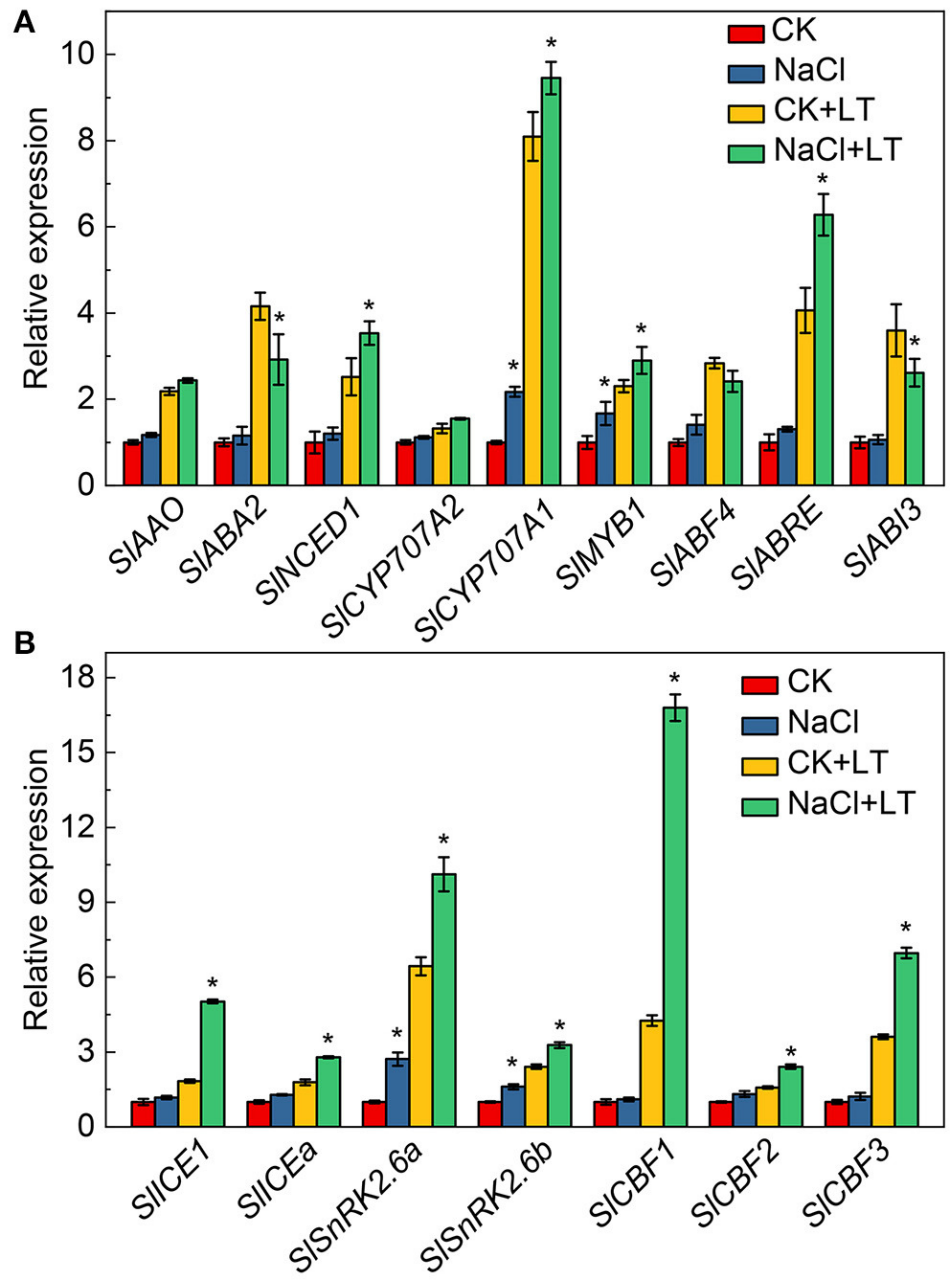
Response of the expression of ABA signaling pathway and cold stress-related genes in tomato leaves to NaCl pretreatment and low temperature stress. The expression of ABA signaling pathway-related genes **(A)** and cold stress-related genes **(B)**. The results are presented as the mean values of three independent biological replicates ± SD, * indicate significant differences between CK and NaCl and between CK+LT and NaCl+LT (*P* < 0.05, Student's *t*-test).

The effects of NaCl pretreatment on the expression of ABA signal transduction and low temperature stress-related genes in tomato leaves were determined. The relative expression levels of the ABA synthesis-related genes *SlNCED1* and the ABA decomposition-related genes *SlCYP707A1* were significantly increased in the NaCl+LT treatment relative to the CK+LT treatment. The relative expression levels of the ABA signal transduction-related genes *SlMYB1* and *SlABRE* were significantly up-regulated in the NaCl+LT treatment compared with the CK+LT treatment of tomato leaves under low temperature stress (Figure 9A). The cold stress-related genes *SlICE1, SlICEa, SlSnRK2.6a, SlSnRK2.6b, SlCBF1, SlCBF2*, and *SlCBF3* were significantly up-regulated under low temperature stress. The relative expression levels of these genes were significantly increased in the NaCl+LT treatment compared with the CK+LT treatment (Figure 9B). These findings indicate that NaCl pretreatment affected the expression of ABA signal transduction and low temperature signaling-related genes in tomato leaves under low temperature stress, which might enhance the resistance of tomato to low temperature stress.

## Discussion

Tomato is a popular vegetable worldwide, and obtaining high-yield and high-quality tomato plants requires a suitable environment. Plants, both in the wild and under controlled conditions (e.g., greenhouses), are often exposed to non-lethal stresses (Zandalinas et al., 2021). These stresses can promote resistance to future lethal stresses and ensure survival in unpredictable environments (Katam et al., 2020). One effective strategy for increasing crop yields in adverse environments that has been successfully applied in recent studies is enhancing the photosynthetic capacity of crops. Crops are often subjected to salt and low temperature stress, and these stresses can lead to stomatal closure, photoinhibition, and reductions in photosynthetic efficiency, which can eventually damage plants. The effects of single stressors on photosynthetic capacity have been extensively studied; by contrast, few studies have examined the effects of multiple stressors on photosynthetic capacity. The results of this study revealed that pretreatment with NaCl and low temperature treatment significantly reduced the photosynthetic carbon assimilation rate and photosystem activity, which is consistent with the results of previous studies; however, we found that NaCl pretreatment could induce tolerance of low temperature stress through photosynthetic acclimation (Figures 1, 2, 4). This acclimation-induced cross-tolerance equips plants with tolerance to multiple stresses following exposure to a specific stimulus; this phenomenon has major agricultural implications given the difficulty of controlling the environments in which many crops are grown (Locato et al., 2018).

Salt and low temperature have deleterious effects on the photosynthetic electron transport and cause excessive light energy to be absorbed by photosynthetic pigments. The severe adversity can substantially exacerbate photoinhibition and induce damage to the photosynthetic apparatus through the production of ROS (Lima-Melo et al., 2019; Yang et al., 2020). The decreases in Fv/Fm and Pm indicate the photoinhibition of PSII and PSI, respectively. Fv/Fm, Pm, PQ size, Y(I), and Y(II) were significantly reduced and Pm, Y(I), and Y(II) were significantly increased in NaCl-pretreated tomato leaves under low temperature stress compared with tomato leaves without pretreated with NaCl. This indicates that NaCl pretreatment can alleviate photoinhibition caused by subsequent low temperature (Figure 4). Previous studies have shown that PSI in many crops, such as *Arabdiopsis*, peanut, and cucumber, tends to experience photoinhibition under low temperature and fluctuating light conditions, which limits crop production (Lima-Melo et al., 2019; Wu et al., 2019; Song et al., 2020; Muhammad et al., 2021; Tan et al., 2021). In contrast to PSII photoinhibition, photoinhibition of PSI cannot be effectively repaired; consequently, recovery of PSI photoinhibition is extremely slow (Kudoh and Sonoike, 2002). The distribution of light energy between photosystems not only determines the amount of excess light energy dissipated by plants in the form of heat but also determines the efficiency of the photochemical reaction. NaCl pretreatment significantly increased Y(I) and Y(II) and significantly decreased Y(NPQ) and Y(ND) of tomato plants suffer subsequent low temperature relative to the plants without NaCl pretreatment. These results might stem from the fact that NaCl pretreatment contributed to induce heat dissipation at both PSI and PSII, which enhanced photosynthetic adaptability of tomato plants and further alleviate the damage caused by low temperature stress (Figures 5, 6).

A range of photoprotective mechanisms can decrease the damage of the PSII and PSI reaction centers, such as chloroplast avoidance movement, dissipation of absorbed light energy as thermal energy (i.e., NPQ), CEF around PSI, and the photorespiratory pathway (Guidi et al., 2019; Bassi and Dall'Osto, 2021). Stomata control the entry of CO_2_ into the cell and the transpiration of leaves and are sensitive to environmental fluctuations. In this study, salt stress caused the stomata to close, and this stomatal adaptation can alleviate the adverse effects of subsequent low temperatures and contributes to the entry of CO_2_, thus maintaining a relatively high net photosynthetic rate (Figure 1). We found that NaCl pretreatment can effectively reduce low temperature-induced chloroplast damage (Figure 3). The degradation of chloroplast proteins is initiated by ROS and involves the action of proteolytic enzymes such as cysteine and serine proteases (Li et al., 2018). Photosynthetic electron transfer is thought to play a major role in controlling chloroplast quality, because of the excessive ROS accumulation caused by photoinhibition can damage chloroplast proteins, and subsequently, the expression of nuclear genes involved in the regulation of the import and degradation of chloroplast proteins were induced through plastid retrograde signaling (Yang et al., 2020).

The CEF-mediated NPQ was increased in NaCl-pretreated tomato plants under low temperature stress, which alleviated photoinhibition and kept photosynthetic performance high (Figures 6, 7). NPQ, which is closely related to CEF, is the most important component of the photoprotection response; the photoprotection of CEF-induced NPQ during the response of plants to stress has been widely studied (Murchie and Ruban, 2020; Bassi and Dall'Osto, 2021). We have previously shown that CEF can modulate linear electron flow and ROS in response to high temperature, and it mainly protects the donor side of PSI under low night temperature stress (Zhang et al., 2014; Lu et al., 2017, 2020a,b). CEF is thought to be closely related to proton gradient production when linear electron transport does not produce sufficient proton gradients across thylakoid membranes. The proton gradient across the thylakoid membrane can induce the protonation of the PSII protein subunit PsbS, which dynamically regulates NPQ (Ikeuchi et al., 2014; Nicol and Croce, 2021). In addition, proton gradients can also down-regulate the electron transfer rate of Cytb6f and activate NPQ through the acidification of the thylakoid lumen. The down-regulation of the electron transport rate through Cytb6f is essential for protecting PSI from damage caused by fluctuations in light (Höhner et al., 2016; Zhou et al., 2022).

Salt stress signal cascades can activate downstream overlapping transduction pathways that enhance the photosynthetic acclimation of plants under low temperature stress, which is consistent with the mechanisms of cross-tolerance (Hossain et al., 2018; Gong et al., 2020). Plants adapt to environmental stresses through photosynthetic acclimation, which involves ROS production, antioxidant defense, ABA, and low temperature signaling pathways. In this study, NaCl stress induced the production of ROS, which activated the oxidative response and the activity of antioxidant enzymes, and these physiological changes could alleviate low temperature-induced damage to plants (Figure 8). In addition, the ABA and low temperature stress signaling were investigated in this study, we found that ABA signal transduction and the low temperature signal pathway contribute to increase the resistance of tomato to low temperature stress (Figure 9). Our results indicates that ROS metabolism, ABA signal transduction, and low temperature signaling pathways can alleviate photoinhibition by activating photoprotection mechanisms, and they also play an important role in regulating salt acclimation-induced cross-tolerance to low temperature stress of tomato. Researches over the past decades have revealed the major functional of apoplastic ROS production and ABA signaling pathway in plants responses to salt and low temperature stress, suggesting that they might be crucial signal molecules mediating cross-tolerance (Van Zelm et al., 2020; Chen et al., 2021). Nevertheless, additional researches are needed to clarify the relationship between stress signaling pathways and photosynthetic acclimation.

## Conclusion

In this study, the photosynthetic mechanism underlying cross-tolerance was characterized through analysis of the photosynthetic performance of tomato plants under NaCl pretreatment and low temperature stress. NaCl treatment reduced the CO_2_ assimilation rate and photochemical reaction efficiency of tomato leaves and induced non-photochemical quenching at PSI and PSII. This, in turn, affected the photosynthetic adaptability of tomato plants and alleviated damage induced by low temperature stress. CEF-mediated photoprotection, stomatal movement, and chloroplast quality maintenance, as well as ABA signal transduction and low temperature stress-related signaling pathways, play a key role in this acclimation process. The results of our study provide new insights into photosynthetic acclimation mechanisms and have implications for environmental management during crop cultivation.

## Data Availability Statement

The original contributions presented in the study are included in the article/Supplementary Material, further inquiries can be directed to the corresponding authors.

## Author Contributions

XY, YL, and TL conceived and designed the experiment. XY and FZ conducted the experiment analyzed the data. XY prepared the manuscript. YZ and JS participated in the experiment and revised the manuscript. MQ participated in the guidance of the experiment. All authors contributed to the article and approved the submitted version.

## Funding

This study was supported by the National Key Research and Development Program of China (2019YFD1000300), the National Natural Science Foundation of China (Grant No. 31772356), China Agriculture Research System of MOF and MARA (CARS-23), the joint fund for innovation enhancement of Liaoning Province (2021-NLTS-11-01), and the support program of young and middle-aged scientific and technological innovation talents (RC210293).

## Conflict of Interest

The authors declare that the research was conducted in the absence of any commercial or financial relationships that could be construed as a potential conflict of interest.

## Publisher's Note

All claims expressed in this article are solely those of the authors and do not necessarily represent those of their affiliated organizations, or those of the publisher, the editors and the reviewers. Any product that may be evaluated in this article, or claim that may be made by its manufacturer, is not guaranteed or endorsed by the publisher.
