# Supplementary material for: NaCl Pretreatment Enhances the Low Temperature Tolerance of Tomato Through Photosynthetic Acclimation
Source: Front Plant Sci. 2022 Jun 13;13:891697. doi: 10.3389/fpls.2022.891697 (PMC10332268; doi:10.3389/fpls.2022.891697)
Supplement: Supplementary file 1 [file Table_1.DOCX]

Supplementary Material

# Supplementary Tables

**Supplementary Table 1. Specific primers used for quantitative real-time polymerase chain reaction in this study.**

| Gene/protein | Primer sequences (5’-3’) |
| --- | --- |
| *SlQUAC1-1* | F-AAACAAAGCAATAAAATCCCAACC  R-CACTTGATAAGGAAACCCTACGAG |
| *SlQUAC1-2* | F-CTCGGCACAAATAGTAATACAAACA  R-CAACTAACAAAGATGCAAAAGCTG |
| *SlSLAC1* | F-ACATAACTCAGGAGACAAAGAGTGC  R-ATAGTTCGGACAGTTAGTCTCGATTA |
| *Slbeta* | F-GATGGATCTAGAGCCTGGTACG  R-CACAATTCTCCGCTTCTTTACG |
| *SlMAP56-1* | F-GCGGAGTTACTTGCGGACAT  R-GCACCTCTGCTTGCATTATACC |
| *SlTUB1-1* | F-GCAATACCGTGCACTTACAGTCC  R-TGAAGGTAGATGCCATTGAGAGAC |
| *SlCAO1* | F- TTATCCACCATTGGCATCTCA  R- TTTCTCCACTATCTACAGCGTCTC |
| *SlCHLD* | F- TGTCACAGAAGATAGATTGATTGG  R- TAGTAGTGGTTTGCATGGATGG |
| *SlCHLG* | F- ATCAAAGGAGCCAAGCAAGA  R- GAAATGGACCAGACATCAACATAC |
| *SlCHLI* | F- TACACCCTTCACTTCTCGTTCC  R-TTCCTCTATCACCCATAATCATCA |
| *SlDVR* | F- GATGTTGTTGTGTCTTGTCTTGC  R- ATCTTGTTCGCTTATCGGCT |
| *SlHEMD* | F- CCGAAGAATGGAAATGACAAG  R- GCTACAGTAACAACGGGAACAGA |
| *SlHEMA1* | F-AGTGTTGGAAGTTTAAGGCTTTT  R- GCCCTCAGTTTCTTGATGGT |
| *SlHEME1* | F- CAAATGGCTATGGCTTTTTCT  R- CAAATGGCTATGGCTTTTTCT |
| *SlHO* | F- TTGCCATTTTTACAACACCTACT  R- ATGACTTCTCTGTCTCTTCCAGAC |
| *SlEEL* | F-TGACACTGGAGGATTTTTTGG  R- GCTTTCATTTTCGCTTCATCA |
| *SlHCAR* | F- GCATGTGCTTTCTTGGGG  R- AGATTAGGAGACAGTGTCGGCT |
| *SlPIF4* | F- ATGGGAATGGGGATGGGT  R- GCTGAGTTTGCTGTGCTGTATG |
| *SlPAO* | F- TTTGTCGCACCTTGTTACTCA  R- ATATCCCCGTCATACACCTTATT |
| *SlNYC1* | F- TGGTTGGTATGGCTTGTGAC  R- TGATTTGGATGGCTTCTTTAGT |
| *SlRCCR* | F- TTCTCACACAATCACTCCCCTC  R- CAACTACCAAGTATGAAATCAACCT |
| *SlMnSOD* | F-GGCACCTACCTCTTCACTCA  R-GGATTGTAATGTGGTCCTGTTGA |
| *SlDHAR* | F-ATGAGGTATCTGCTGCTGATTTG  R-CTTTTAGTGCCCGCGTGTTG |
| *SlMDHAR* | F-ACACACGCACACGTATGACTATTT  R-TCAGGACTCCCACTTTCAAGA |
| *SlPOD* | F-TTGTCCCCAGACTGTATCTTGTG  R-TGTTATTTGCTGCTGTTTTGCTT |
| *SlCAT* | F-GGTGCTAGTGCCAAAGGGTT  R-TCGGGATGATGAGAGAAAAAAT |
| *SlGR* | F-GGCTACATCTTTGAGCTCACC  R-CGGAGAGGCTTGATAGGGTT |
| *SlAAO* | F- GTGAGTTTGGTTTTGGCAGTT  R- GCATGGAAACCGGCAAACCT |
| *SlABA2* | F- ACTTGCCTCCTGAACAAAGG  R- CTTAGCAACATCCTGAGCCA |
| *SlNCED1* | F-AGGCAACAGTGAAACTTCCATCAAG  R-TCCATTAAAGAGGATATTACCGGGGAC |
| *SlCYP707A2* | F- AGTACAGGTGGTCTATGGTGGG  R- GTTGATGATGTTTTGAGAGAGAGTTT |
| *SlCYP707A1* | F- ATCACAACCCAGAGTTCTTTCCT  R- CAGATCCTACCACTTCCCACC |
| *SlMYB1* | F- GAATTTTCCATCATTGAGGGTTT  R- TCACTTGAGTAGTTTCCAGGGTT |
| *SlABF4* | F- GAAGAGTCTCAAGCGTTGT  R- TGATCCTCCAGTCCCAC |
| *SlABRE* | F- TCCAGCACTCAACTCT  R- TGGCTCCTAAACCTAC |
| *SlABI3* | F- TTGATACGAGGGGTAAAAGTGAG  R- AACAGCTTGTGCAAATGGAGAT |
| *SlICE1* | F- TCCATCCTCTGACACCGACT  R- CCTTCCCTGACCCTCACTTC |
| *SlICEa* | F- TTCTTGCTGGTGGTGGGT  R- AAAAAGGGTTGGTTGTGCTC |
| *SlSnRK2.6a* | F- AATCAATCACAGGTCGCTGAGGCATCC  R- GTTGCTGGAAGAAGAAGCGAGCCTGAG |
| *SlSnRK2.6b* | F- TGCTGGTCGTTTTAGCGAGGATGAG  R- GGAGGTTAGGAATCAACTTGTGGCA |
| *SlCBF1* | F- GAGTCGGAAGAAGTTTCAGG  R- TGTAGGCATCAGTTTCCAC |
| *SlCBF2* | F- TTCGATCGGAAGAAGTTTCA  R- CAAGTAATCCTGGCATGGAA |
| *SlCBF3* | F- TGCCGGGTTTACTTACGAAT  R- TCAGCTTCCACATGATCTCC |
